# Telmisartan improves cardiac fibrosis in diabetes through peroxisome proliferator activated receptor δ (PPARδ): from bedside to bench

**DOI:** 10.1186/s12933-016-0430-5

**Published:** 2016-08-12

**Authors:** Wei-Ting Chang, Juei-Tang Cheng, Zhih-Cherng Chen

**Affiliations:** 1 Department of Cardiology, Chi Mei Medical Center, 901, Zhonghua Road, Yongkang District, Tainan, Taiwan, ROC; 2 Department of Medical Research, Chi Mei Medical Center, Tainan, Taiwan; 3 Department of Pharmacy, Chia Nan University of Pharmacy and Science, Tainan, Taiwan

**Keywords:** Telmisartan, Diabetic cardiomyopathy, PPARδ, STAT3

## Abstract

**Background:**

Despite the known risk of diabetes-induced cardiac fibrosis, less is known about whether diabetes causes an altered cardiac phenotype independent of coronary atherosclerosis. Peroxisome proliferator-activated receptor δ (PPARδ), a versatile regulator of metabolic homeostasis, may be a potential therapeutic target. Herein we investigated the effectiveness of telmisartan, a unique angiotensin receptor blocker that increases PPARδ expression, in improving left ventricular remodeling in diabetic humans and rats.

**Methods:**

In this longitudinal, prospective study, we enrolled 15 diabetic patients receiving telmisartan (20 mg/day) for 12 weeks. After treatment, strain was measured and compared with the baseline value. Using streptozotocin to induce type 1 diabetes rat model, we measured PPARδ expression and downstream targets.

**Results:**

After treatment with telmisartan, both longitudinal and circumferential strains improved in diabetic patients. Compared with that of controls, the diabetic rat heart developed significant fibrosis, which markedly decreased after treatment with telmisartan (30 mg/kg/day, orally) for 7 days. After incubation with 30 mM glucose, rat cardiomyocytes showed a significant down-regulation of PPARδ. Interestingly, the increased expression of fibrosis-associated proteins, including signal transducer and activator of transcription 3 (STAT3) was attenuated by the co-incubation of GW0742, a PPARδ agonist. By knockdown or inhibition of STAT3, the hyperglycemia related high expression of fibrosis associated targets was reversed. Independent from the hyperglycemic incubation, STAT3 over-expression led to similar results. Conversely, in the presence of GSK0660, a PPARδ inhibitor, the protective effects of telmisartan were diminished.

**Conclusion:**

Telmisartan improved the hyperglycemia-induced cardiac fibrosis through the PPARδ/STAT3 pathway.

**Graphical abstract:**
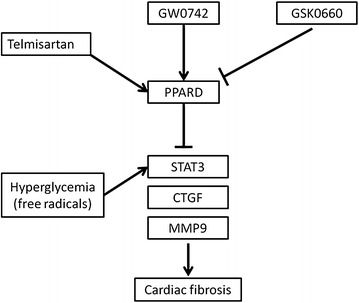
Summary of the mechanism of telmisartan’s effect on the suppression of hyperglycemia-induced cardiac fibrosis through PPARδ instead of the AMPK pathway.* PPARδ* peroxisome proliferator-activated receptor δ, * STAT3* signal transducer and activator of transcription 3,* CTGF* connective tissue growth factor,* MMP9* matrix metallopeptidase 9

## Background

The prevalence of diabetes has continued to increase, and its complications, including cardiac fibrosis-induced myocardial dysfunction and chronic heart failure, are threats to the health of millions of people [1, 2]. However, little is known about whether diabetes causes an altered cardiac phenotype independent of coronary atherosclerosis. To detect myocardial dysfunction at an early stage, instead of histological evidence, imaging methods, such as speckle-tracking echocardiography (STE), can be used to unmask subtle changes in the cardiac function of patients with systemic conditions [3]. Telmisartan, an angiotensin receptor blocker (ARB), lowers not only blood pressure but also insulin resistance [4]. Different from other ARBs, telmisartan did not increase the incidence of diabetes and conversely, ameliorated atrial remodeling as well as reduced susceptibility to atrial arrhythmia [5, 6]. In a study using STE, telmisartan was observed to have beneficial effects on left ventricular (LV) structure in a hypertensive patient [7, 8]. More recently, it was also found to ameliorate LV-remodeling in post-infracted or doxorubicin-induced cardiomyopathy by activating peroxisome proliferator-activated receptors (PPARs) [9–12]. Among PPARs, PPARδ is widely observed in diabetic disorders, likely because cardiomyocyte-restricted PPARδ deletion causes cardiac hypertrophy [13–15]. Previous studies have indicated that inhibitors of reactive oxygen species (ROS) production or mitogen-activated protein kinase (MAPK) activation are involved in the reduction of cardiac PPARδ expression in response to hyperglycemia in hepatocytes and adipocytes [16]. Signal transducer and activator of transcription 3 (STAT3), a pivotal mediator in cardiac hypertrophy, was also found to be involved in PPARβ/δ-regulated insulin resistance in acute liver disease [17, 18]. Nevertheless, the mechanisms of improvement of cardiac remodeling through ARBs have not been fully elucidated in diabetic patients. In this study, we aimed to investigate the regulatory role of PPARδ in diabetes-induced cardiac fibrosis under telmisartan treatment.

## Methods

### Objective

Fifteen patients at the Chi-Mei Medical Center who were newly diagnosed with diabetes according to the definition of the World Health Organization were initially included between June 2014 and March 2015. In addition, 10 age- and gender-matched controls were enrolled. The informed consent was obtained from each patient and the study was conducted according to the recommendations of the Declaration of 1975 Helsinki on Biomedical Research involving human subjects and was approved by the local Ethics Committee (IRB: 10307-003). Written informed consent was obtained from each participant. All participants underwent echocardiographic evaluation before and after 3 months of telmisartan therapy.

### Echocardiography

Standard echocardiography was performed (Vivid E9; GE Vingmed Ultrasound AS, Horten, Norway) with a 3.5-MHz multiphase-array probe in accordance with the recommendations of the American Society of Echocardiography [19]. LV ejection fraction (LVEF) was measured using the biplane Simpson’s method. In addition, LV diastolic function-associated parameters including isovolumic contraction time (IVCT), isovolumic relaxation time (IVRT), ejection time (ET), deceleration time, transmitral and tricuspid early filling velocity (E) to atrial velocity (A) ratio were measured. Tissue Doppler imaging values of the right and left ventricles were obtained from the apical four-chamber view using a sample volume placed at the lateral corner of the tricuspid annulus and anterior and lateral sections of the mitral annulus. Peak systolic annular velocity (S′) and early (e′) and late (a′) annular diastolic velocities were measured. In addition, myocardial performance index, also known as the Tei index, was calculated as (IVCT + IVRT)/ET.

### STE analysis for deformation

Standard apical four-chamber and short-axis views were recorded in digital loops for deformation analysis of the LV [3]. The images were acquired with frame rates of 70–90 frames/s and stored for three cycles. The images were analyzed off-line using EchoPAC 9.0 computer software (GE-Vingmed Ultrasound AS). As described previously, we measured the LV peak systolic global longitudinal strain in the apical views. In addition, LV circumferential strain was also obtained from the short-axis view at the papillary muscle level.

## Materials

GW0742 (a specific PPARδ agonist) and GSK0660 (a specific PPARδ antagonist) were purchased from Santa Cruz Biotechnology, Inc. (Santa Cruz, CA, USA). The antibodies, including STAT3 and connective tissue growth factor (CTGF), were purchased from Abcam (Cambridge, MA, USA) and matrix metallopeptidase 9 (MMP9) was from Minipore.

### Animals

Male Sprague–Dawley rats, weighing 200–250 g, were obtained from the Animal Center of National Cheng Kung University Medical College. All experiments were performed under anesthesia with 3 % isoflurane, and all efforts were made to minimize suffering. The animal experiments were approved and conducted in accordance with local institutional guidelines for the care and use of laboratory animals in Chi-Mei Medical Center (No. 100052307) and conformed to the Guide for the Care and Use of Laboratory Animals. At the end of the experiments, hearts were excised from isoflurane-euthanized mice, washed in phosphate-buffered saline (PBS), fixed overnight in 4 % paraformaldehyde, and embedded in paraffin. After serial sectioning of hearts, 5-μm sections were stained with Masson trichrome for the quantification of myocardial fibrosis.

### Streptozotocin (STZ)-induced type 1-like diabetic rats

Diabetic rats were induced by intravenously injecting STZ at 65 mg/kg (Sigma-Aldrich Inc., USA) into fasting rats as described previously. The animals were considered diabetic if they had a plasma glucose concentration over 350 mg/dL. Diabetic rats received either telmisartan (30 mg/kg/day, orally) or metformin (100 mg/kg/day, orally). To evaluate the role of PPARδ in the effects of telmisartan, some rats were treated with the PPARδ antagonist GSK0660 (1 mg/kg/day, intraperitoneally) 30 min before the telmisartan treatment. Each condition includes eight rats.

### Cell cultures

H9C2 cells were used in the experiments after 3–4 days in culture. To mimic the hyperglycemic status, cells were incubation with 30 mM glucose for 24 h and then changed to a glucose free medium. In alternative groups, H9C2 cells were subsequently treated with GW0742 (10^−6^ M) for 30 min, washed twice with PBS, and removed by trypsinization. The cells were then collected and subjected to a protein expression assay. Cells were additionally treated with GSK0660 (10^−6^ M) for 30 min before the GW0742 treatment.

### Western blotting analysis

Protein was extracted from tissue homogenates and cell lysates using ice-cold radioimmunoprecipitation assay buffer supplemented with phosphatase and protease inhibitors (50 mM sodium vanadate, 0.5 mM phenylmethylsulfonyl fluoride, 2 mg/mL aprotinin, and 0.5 mg/mL leupeptin). Protein concentrations were determined using a Bio-Rad protein assay (Bio-Rad Laboratories, Inc., Hercules, CA, USA). Total proteins (30 mg) were separated by sodium dodecyl sulfate–polyacrylamide gel electrophoresis (10 % acrylamide gel) using a Bio-Rad Mini-Protein II system. The protein was transferred to expanded polyvinylidene difluoride membranes (Pierce, Rockford, IL, USA) with a Bio-Rad Trans-Blot system. After transfer, the membranes were washed with PBS and blocked for 1 h at room temperature with 5 % (w/v) skimmed milk powder in PBS. The manufacturer’s instructions were followed for the primary antibody reactions. Blots were incubated overnight at 4 °C with an immunoglobulin-G polyclonal rabbit antimouse antibody (Affinity BioReagents, Inc., Golden, CO, USA) (1:500) in 5 % (w/v) skimmed milk powder dissolved in PBS/Tween 20 (0.5 % by volume) to bind the target proteins such as PPARδ. The blots were incubated with goat polyclonal antibody (1:1000) to bind the actin that served as the internal control. After the removal of the primary antibody, the blots were extensively washed with PBS/Tween 20 and then incubated for 2 h at room temperature with the appropriate peroxidase-conjugated secondary antibody diluted in 5 % (w/v) skimmed milk powder and dissolved in PBS/Tween 20. The blots were developed by autoradiography using an ECL-Western blotting system (Amersham International, Buckinghamshire, UK). The immunoblots of cardiac troponin I (28 kDa) and phosphotroponin I (28 kDa) were quantified using a laser densitometer.

### Small interfering RNA (siRNA) transfection assay

Double-stranded siRNA sequences targeting STAT3 mRNA were obtained from Sigma Biotechnology. The cells were cultured in 100-mm-well plates in medium. Transfection with siRNA was carried out with transfection reagent (PureFection™; System Biosciences, Mountain View, CA, USA). Specific silencing was confirmed by immunoblotting with cellular extracts after the transfection.

### Reverse transcription polymerase chain reaction (RT–PCR)

Total RNA of rat LV was isolated following the protocol described previously. Two hundred nanograms of total RNA was reverse transcribed into cDNA with random hexamer primers (Roche Diagnostics, Mannheim, Germany). The PPARδ gene expression was measured using RT–PCR. Expression of hydroxymethylbilane synthase (HMBS), a housekeeping gene, was analyzed for normalization and quality control. We used the following primers: PPARδ forward 5′-aagacaaacccacggtaaagg-3′ and reverse 5′-catgactgacccccacttg-3′, MMP9 forward 5′-tcgtggctctaaacctgacc-3′ and reverse 5′-gagctgtcggctgtggtt-3′, CTGF forward 5′-atgctgtgaggagtgggtgt-3′ and reverse 5′-ggccaaatgtgtcttccagt-3′, and HMBS forward 5′-tgccctggagaagaatgaag-3′, and reverse 5′-cagcatcatgagggttttcc-3′. PCR amplification of these genes was carried out with 20 ng cDNA, 200 nM forward and reverse primers, and TaqMan Master Mix (Roche Diagnostics) in a final volume of 10 μL. PCRs were run in a LightCycler 2.0 (Roche Diagnostics) for 45 cycles, with each cycle consisting of denaturation for 15 s at 95 °C, primer annealing for 15 s at 55 °C, extension for 30 s at 72 °C, and cooling 30 s at 40 °C.

### Statistical analysis

Differences among normal control subjects and diabetic patients before and after telmisartan treatment were compared using Student’s *t* tests for normally distributed continuous variables, nonparametric tests for non-normally distributed continuous variables, and χ^2^ tests for categorical variables. Group differences were analyzed using analysis of variance. Factors with *p* < 0.1 based on the univariate analyses were included in the multivariate logistic regression analyses. A *p* < 0.05 was considered significant. All analyses were performed using SPSS version 18 for Windows (SPSS Inc., Chicago, IL, USA).

## Results

### Telmisartan improved cardiac remodeling in diabetic patients

**Fig. 1:**
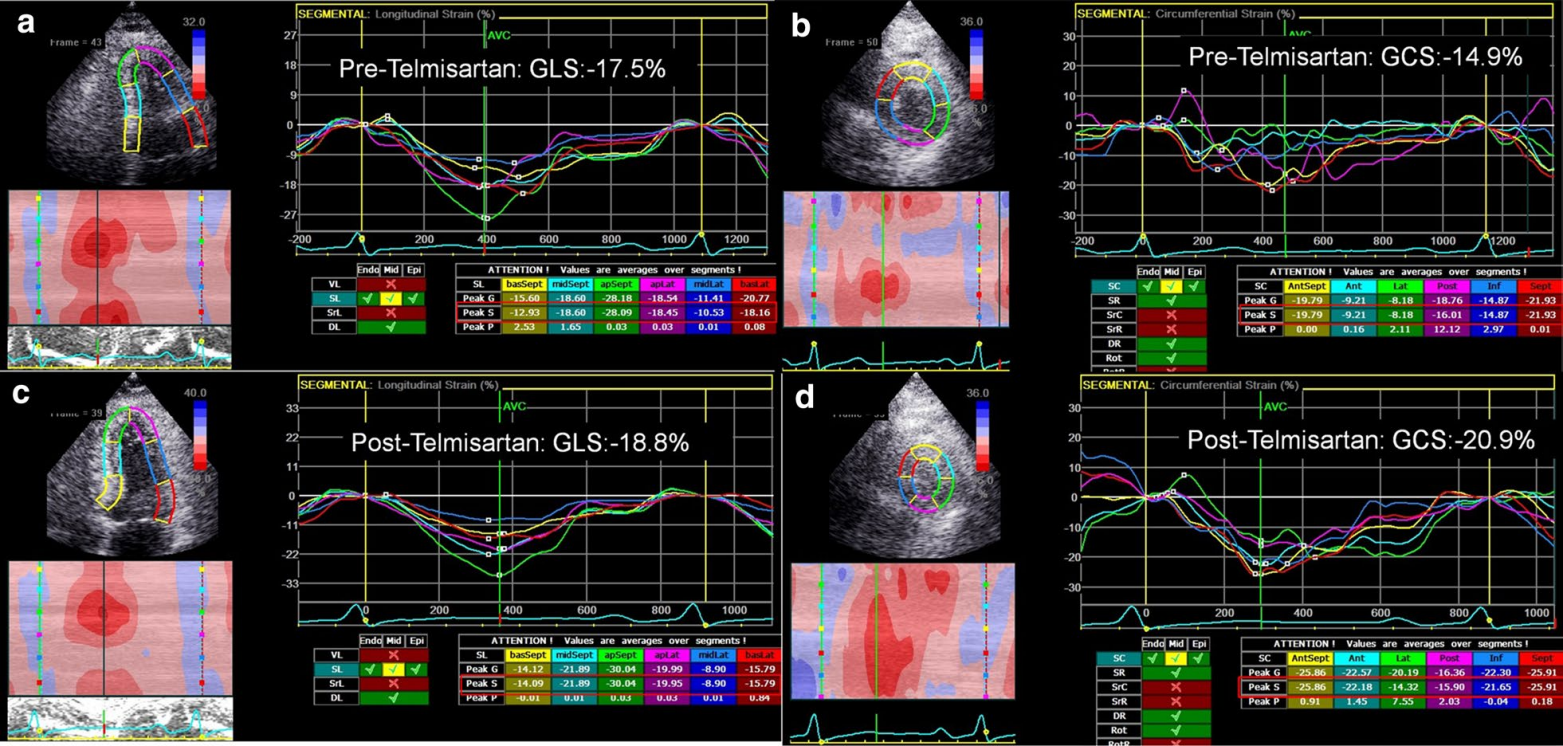
An illustration of speckle-tracking imaging analysis in diabetic patients. **a** The longitudinal and **b** circumferential strains in diabetic patients before telmisartan treatment. **c**, **d** The improvement of longitudinal **c** and circumferential (**d**) strains in diabetic patients after telmisartan treatment. *GLS* systolic global longitudinal strain, *GCS* systolic global circumferential strain

Among the control group and diabetic patients before and after telmisartan treatment, no significant differences in clinical characteristics were observed except for blood glucose and hemoglobin A1c. All diabetic patients received one or two types of oral antidiabetic agents (Table 1). There were no differences in blood pressure, LVEF, or E/A (Table 2). However, compared with normal control subjects, the mean peak systolic strains in the longitudinal and circumferential directions (Fig. 1) and tissue Doppler-derived e′ were more impaired in the patients with diabetes. Furthermore, after 3 months of telmisartan treatment, the mean peak systolic strains in both the longitudinal and circumferential directions improved compared with baseline values.

**Table 1:** Clinical characteristics of the normal control and diabetic patients before and after 3 months of telmisartan treatment

	Normal control (n = 10)	Baseline (n = 15)	After telmisartan (n = 15)	*p* value
Age (years)	52.2 ± 8.5	55.3 ± 8.8	–	0.25
Male (%)	6 (60)	11 (73.3)	–	0.12
Body height (cm)	165.8 ± 7.4	163.9 ± 8.7	–	0.72
Body weight (kg)	68.6 ± 7.1	70.8 ± 10.6	–	0.81
Heart rate (beats/min)	69.8 ± 7.6	73.5 ± 8.24	72.38 ± 11.74	0.64
Systolic blood pressure (mmHg)	120.2 ± 12.9	129.6 ± 17.1	122.1 ± 14.4	0.06
Diastolic blood pressure (mmHg)	83 ± 8.9	82.6 ± 7.7	85.2 ± 13.9	0.43
Preprandial serum glucose (mg/dL)	92.7 ± 5.6	122 ± 15.8	105.3 ± 10.6	*0.01*
HbA1c (%)	5.6 ± 0.9	7.8 ± 1.2	7.2 ± 0.8	*0.02*
Creatinine clearance rate (ml/min)	108.7 ± 45.8	83.7 ± 24.5	95.8 ± 24.6	0.29
Triglyceride (mg/dL)	122.6 ± 51.3	129 ± 6.2	–	0.59
Cholesterol (mg/dl)	178.7 ± 28.8	178.6 ± 32.3	–	0.67

Data are expressed as mean ± standard deviation or median (interquartile range). Italic values indicate significance

*HbA1c* hemoglobin A1c

**Table 2:** Conventional and speckle-tracking echocardiographic parameters of normal control and diabetic patients before and after 3 months of telmisartan treatment

	Normal control (n = 10)	Baseline (n = 15)	After telmisartan (n = 15)	*p* value
LVMI (g/m^2^)	83.3 ± 29.1	84.5 ± 29.8	85.1 ± 30.2	0.63
LVIDd (cm)	4.2 ± 0.8	4.1 ± 0.7	4.1 ± 1.4	0.52
LVEF (%)	70.5 ± 6.4	68.1 ± 4.5	69.3 ± 15.7	0.38
E (cm/s)	76.5 ± 18.3	79.5 ± 12.3	82.7 ± 18.3	0.72
E/A	1.0 ± 0.3	1.0 ± 0.2	0.9 ± 0.6	0.06
e′ (cm/s)	10.3 ± 2.8	8.2 ± 1.8	9.3 ± 2.7	*0.01*
IVRT (ms)	94.7 ± 23	96.6 ± 13.1	90.7 ± 21.5	0.06
DT (ms)	200.5 ± 58.6	184.1 ± 52.2	198.4 ± 43.7	0.93
MPI	0.4 ± 0.2	0.5 ± 0.1	0.5 ± 0.1	0.35
GLS (%)	−20.2 ± 6.7	−16.4 ± 5.2	−18.1 ± 6.2	*0.03*
GCS (%)	−22.2 ± 7.8	−14.7 ± 8.6	−20.8 ± 6.8	*0.01*

Data are expressed as mean ± standard deviation. Italics indicate significance

*LVMI* left ventricular mass index, *LVIDd* left ventricular interior dimension at end diastole, *LVEF* left ventricular ejection fraction, *IVRT* isovolumic relaxation time, *DT* deceleration time, *E/A* transmitral valve E to A velocity ratio, *E/eʹ* mitral early filling velocity to early diastolic mitral annular velocity ratio, *MPI* myocardial performance index, *GLS* peak systolic global longitudinal strain, *GCS* peak systolic global circumferential strain

### Telmisartan decreased diabetes-induced cardiac fibrosis in STZ rats through the PPARδ pathway

**Fig. 2:**
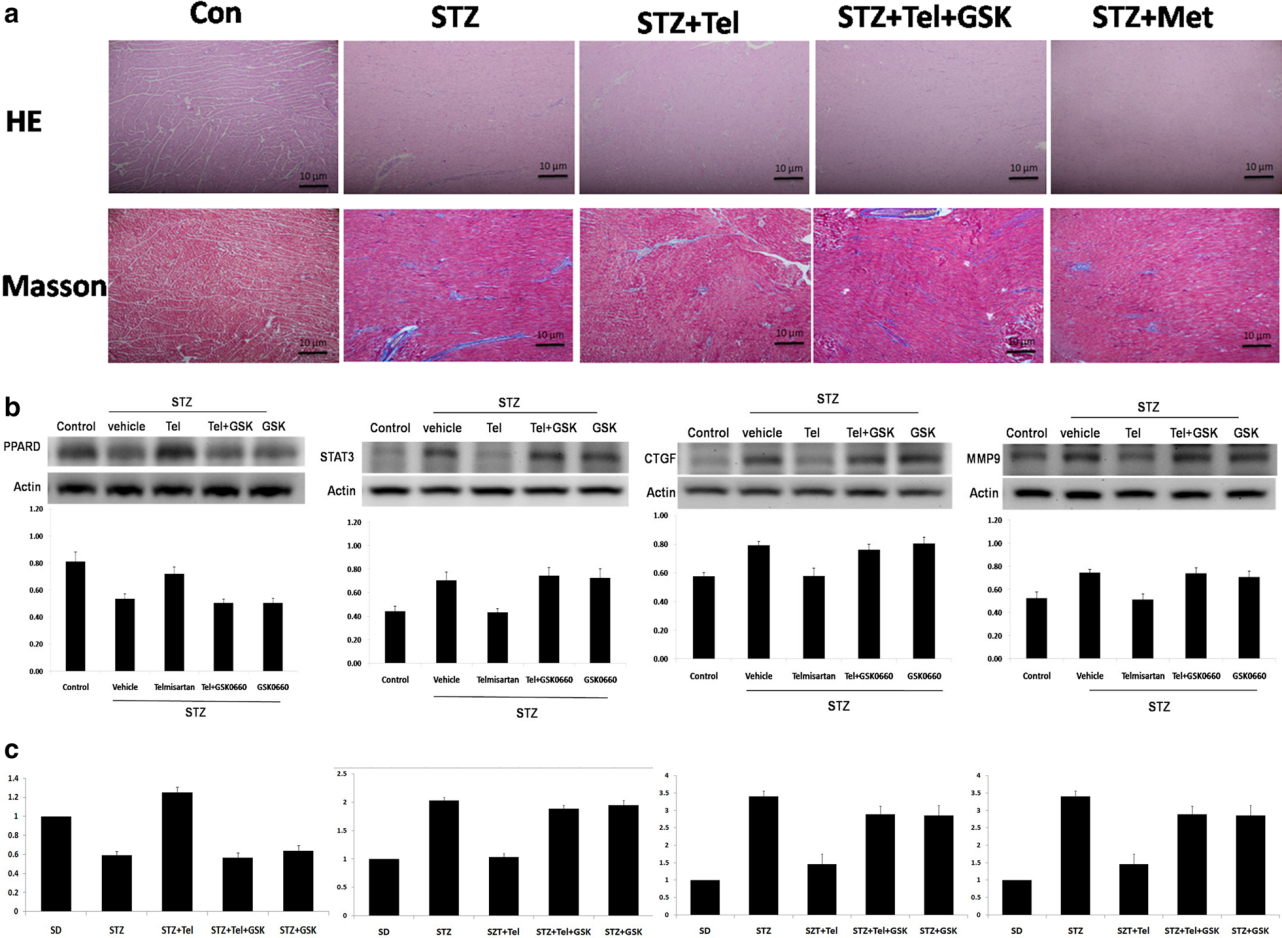
Telmisartan decreased diabetes-induced cardiac fibrosis in STZ rats via PPARδ. **a** Hematoxylin and eosin and Masson trichrome staining of control (Sham) rat hearts, STZ-treated rat hearts, and STZ-treated rat hearts treated with telmisartan, telmisartan plus GSK0660 (a *PPARδ antagonist*), or metformin. **b** The expression of PPARδ, STAT3, CTGF, and MMP9 proteins in the variously treated rat hearts. **c** The relative expression of PPARδ, STAT3, CTGF, and MMP9 in variously treated rat hearts. *STZ* streptozotocin, *Tel* telmisartan, *PPARδ* peroxisome proliferator-activated receptor δ, *STAT3* signal transducer and activator of transcription 3, *CTGF* connective tissue growth factor, *MMP9* matrix metallopeptidase 9

Masson trichrome staining revealed a significant increase of fibrotic intensity in the STZ-induced diabetic rat hearts compared with the control hearts. Notably, the cardiac fibrosis in the diabetic rats ameliorated significantly post treatment with telmisartan but not under the treatment of metformin, an AMP-activated protein kinase (AMPK) pathway activator. Conversely, additional treatment with the PPARδ antagonist GSK0660 diminished the improvement (Fig. 2a). Telmisartan reversed the STZ-induced downregulation of PPARδ. Compared with the control rats, the expression of PPARδ was significantly attenuated in the STZ-treated rat hearts but increased after the telmisartan treatment. Notably, once PPARδ was blocked by GSK0660, the effect was extinguished. Notably, the fibrosis-associated proteins, including CTGF, MMP9, and STAT3, were significantly upregulated in STZ-treated rat hearts but decreased under the treatment of telmisartan via the PPARδ pathway (Fig. 2b). Likewise, a similar finding was observed regarding the relative expression of PPARδ and the downstream proteins using RT–PCR (Fig. 2c).

### Effect of PPARδ on cardiac fibrosis in high-glucose–treated cardiomyocytes

**Fig. 3:**
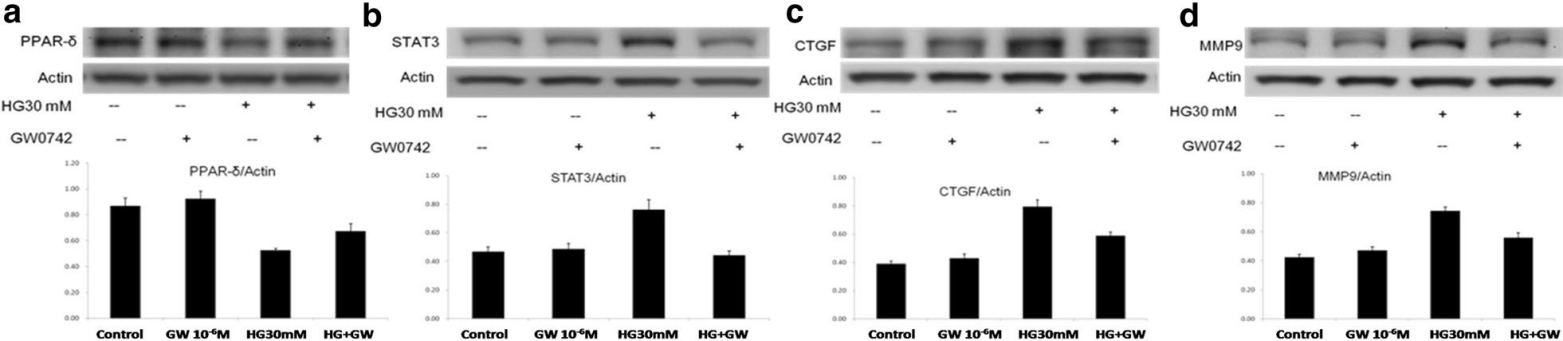
Expression of fibrosis-associated proteins in cardiomyocytes Cardiomyocytes in hypoglycemic conditions were treated with GW0742 (a PPARδ agonist). **a** PPARδ; **b** STAT3; **c** CTGF; and **d** MMP9. *PPARδ* peroxisome proliferator-activated receptor δ, *STAT3* signal transducer and activator of transcription 3, *CTGF* connective tissue growth factor, *MMP9* matrix metallopeptidase 9

After incubation with 30 mM glucose, rat cardiomyocytes showed a significant downregulation of PPARδ. Consequently, the expression of fibrosis-associated proteins, including STAT3, CTGF, and MMP9, increased. Nevertheless, co-treatment with GW0742, a PPARδ agonist, partially rescued the downregulation of PPARδ and additionally attenuated the upregulation of STAT3, CTGF, and MMP9. Notably, without the condition of hyperglycemia, GW0742 failed to increase the expression of PPARδ or to affect the subsequent fibrosis-associated proteins (Fig. 3).

### The interplay between PPARδ and STAT3 with respect to cardiac fibrosis

**Fig. 4:**
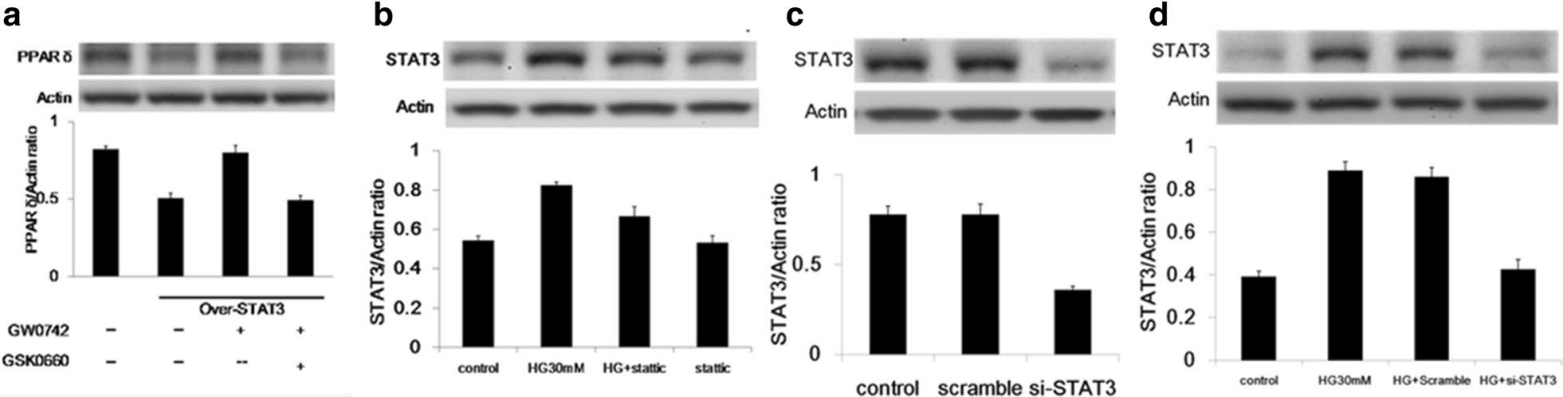
Protein expression of PPARδ and STAT3 in cardiomyocytes. **a** Protein expression of PPARδ in STAT3-overexpressing cardiomyocytes treated with GW0742 or GSK0660. **b** Protein expression of STAT3 in cardiomyocytes treated with Stattic, an inhibitor of STAT3 under hyperglycemic conditions. **c** Downregulation of STAT3 in cardiomyocytes treated with si-STAT3. **d** Protein expression of STAT3 in the STAT3 knockdown cardiomyocytes in high-glucose conditions. *PPARδ* peroxisome proliferator-activated receptor δ, *STAT3* signal transducer and activator of transcription 3, *si* small interfering

Alternatively, the overexpression of STAT3 in cardiomyocytes resulted in a significant downregulation of PPARδ (Fig. 4a). Co-treatment with GW0742 was sufficient to reactivate PPARδ, but the effect was attenuated by the PPARδ inhibitor GSK0660. Conversely, the upregulation of STAT3 induced by high glucose was decreased by treatment with *Stattic*, a small-molecule inhibitor of STAT3 dimerization (Fig. 4b). Instead, of external inhibition of STAT3, internal knockdown of STAT3 by siRNA also directly reversed the hyperglycemia-related high expression of CTGF, STAT3, and MMP9 (Fig. 4c, d).

## Discussion

In the present study, we found that (1) telmisartan improved cardiac remodeling in patients with diabetes; (2) the development of cardiac fibrosis in the diabetic rat was markedly decreased after telmisartan treatment through the PPARδ pathway; (3) the regulatory mechanism of PPARδ on the hyperglycemia-induced fibrosis was dependent on the activation of STAT3. To the best of our knowledge, this is the first multidisciplinary study coordinating the clinical imaging, histologic evidence, and molecular mechanisms to investigate the therapeutic potentials of a new target, PPARδ, in protection from diabetes-induced cardiac fibrosis.

### Diabetes-related cardiac fibrosis

Diabetes has emerged as a major threat to worldwide health, and the incidence continues to increase each year [1]. The importance of diabetic cardiomyopathy, i.e., congestive heart failure in diabetic patients in the absence of discernible coronary artery disease (valvular disease or hypertension), has received more attention in recent decades [1]. Diabetic cardiomyopathy is characterized by myocyte loss and myocardial fibrosis, leading to decreased elasticity and impaired contractile function [1, 20]. Regarding its pathophysiology, the main theory focused on the accumulation of extracellular matrix proteins [1, 21]. Among them, MMP9 and CTGF are crucial in virtually all diabetes-induced fibrotic pathology, including nephritic, retinal, and cardiac fibrosis [21, 22]. In addition, the conversion of energy catabolism in mitochondria and the subsequent ROS production were also proposed to contribute to the structural and functional abnormalities [22, 23]. However, the direct regulator of the development of diabetic cardiomyopathy remains unknown.

### The role of telmisartan in cardiac remodeling

To detect myocardial dysfunction at an early stage, instead of histological evidence, imaging, such as STE, can be used to unmask subtle changes in the cardiac function of patients with systemic conditions such as cancer or diabetes mellitus [3]. In a previous study using STE, telmisartan was found to have beneficial effects on LV structure and function in patients with hypertension [7, 8]. Notably, telmisartan shows not only antihypertensive but also several pleiotropic effects that interact with metabolic pathways to ameliorate cardiac remodeling by activating PPARs [4]. In the work of Mikami and colleagues, telmisartan activated endogenous PPARδ and played an antifibrotic role in human mesangial cells [24]. In skeletal muscle, telmisartan was shown to enhance running endurance through activation of the PPARδ pathway [25]. More specifically, using PPARδ knockout mice, the effect of telmisartan on insulin signaling and glucose uptake was validated to involve PPARδ or phosphatidylinositol-3 kinase but not PPARγ and PPARα [26, 27]. In our study, the cardiac fibrosis in the diabetic rats was significantly ameliorated by telmisartan but not metformin, an AMPK pathway activator [28]. By blocking PPAR-δ, the telmisartan-related improvement of cardiac fibrosis was extinguished, and consequently, the downstream fibrosis-associated proteins were significantly reactivated [26]. Taken together, telmisartan reversed the hyperglycemia-induced cardiac fibrosis through the PPARδ pathway instead of the AMPK pathway. Interestingly, our study also indicated that enhancing the expression of PPARδ attenuated the upregulation of subsequent fibrosis-associated proteins in cardiomyocytes in hyperglycemic conditions. This result implied that the effect of PPARδ on the cardiomyocytes may be independent from telmisartan.

### The effects of PPARδ on hearts

PPARδ has emerged as a versatile regulator of metabolic and inflammatory homeostasis [13, 14, 29]. Deletion of cardiac PPAR-δ is accompanied by decreased contraction, lowered cardiac output, and decreased contraction, which leads to cardiac failure [13]. In our previous study, we also demonstrated that activation of PPARδ using the selective agonist GW0742 enhanced cardiac contractility in isolated hearts and the hemodynamics in rats without altering heart rate [14]. Regarding the mechanism of how PPARδ ameliorates cardiac fibrosis, a previous study indicated that the PPARβ/δ agonist inhibited STAT3 activation and insulin resistance in human liver cells [17]. Likewise, activation of PPARβ/δ ameliorated insulin signaling by inhibiting STAT3 in interleukin-6-stimulated adipocytes [26]. In the work of Serrano-Marco and colleagues, activation of PPARβ/δ with an agonist prevented induction of the transcription factor STAT3, which results in the prevention of insulin resistance in adipose tissue [18]. Nevertheless, the details remain unknown.

STAT3 is fundamental for physiological homeostasis and is involved in multiple post-translational modifications, including proliferation, differentiation, and metabolism in cardiomyocytes, fibroblasts, and various inflammatory cells [30]. Downregulation of STAT3 is sufficient to induce dilated and adverse remodeling after myocardial infarction [30, 31]. However, overexpression of STAT3 also results in cardiac hypertrophy [32]. Interestingly, STAT3 also contributes to insulin resistance and glucose homeostasis [33]. Inhibition of mammalian target of rapamycin was found to protect against reperfusion injury in the diabetic heart through STAT3 signaling [30, 34]. In agreement with our findings, losartan, also an ARB, reduced myocardial interstitial fibrosis in diabetic cardiomyopathy rats by inhibiting the Janus kinase/STAT signaling pathway [4]. However, in contrast to above finding, we found that the overexpression of STAT3 directly downregulated PPARδ independent from the stimulation of hyperglycemia [31]. This result implied the importance of STAT3 in myocardial fibrosis and its potential role in homeostasis along with PPARδ. To conclude, telmisartan works through PPARδ, instead of the AMPK pathway, to suppress the hyperglycemia-enhanced expression of STAT3/CTGF/MMP9 and the subsequent cardiac fibrosis (Graphic abstract).

There are some limitations of this study. First, Telmisartan works not only in PPARδ but PPAR gamma [35–39]. However, PPARγ activator, like Thiazolidinediones, is associated with side effects such as edema and body weight gain [40]. Therefore, using a PPARδ specific ligand, GW0742, can activate the protective effects from hypertriglyceridemia and insulin resistance as what PPARα and PPARγ do but alternatively prevents the sequel of edema and fluid retention induced by PPARγ [14, 18]. Therefore, we suggested using a PPARδ specific ligand instead of telmisartan in the future clinical studies. Second, according to our findings, it is concluded that the myocardium protective effect of PPARδ in diabetic cardiomyopathy is regulated by STAT3 and we believed that it is indirectly through ROS reduction. As we know, ROS plays an integral role in dys-regulation of diabetic myocardium remodeling and notably, a number of anti-oxidant and antiapoptotic genes are upregulated by STAT3 [41]. In another aspect, agonist-activated PPARδ suppressed the generation of Angiotensin II-triggered ROS with a concomitant reduction in DNA damage [6, 17]. Taken together, ROS is believed to be involved in the relationship between PPARδ and STAT3 and we will focus on it in the future studies.

## Conclusion

These results indicate that telmisartan activates endogenous PPARδ and may prevent CTGF/MMP9-induced fibrotic changes by upregulating STAT3 expression in a hyperglycemic environment. These findings potentially elucidate the beneficial effects of telmisartan on LV structural and functional restoration.

## Abbreviations

ARB: angiotensin receptor blocker
CTGF: connective tissue growth factor
ET: ejection time
DT: deceleration time
E/A: transmitral and tricuspid early filling velocity to atrial velocity ratio early (e′) and late (a′) annular diastolic velocities
HMBS: hydroxymethylbilane synthase
IVCT: isovolumic contraction time
IVRT: isovolumic relaxation time
LVEF: left ventricular ejection fraction
MAPK: mitogen-activated protein kinase
MMP9: matrix metallopeptidase 9
PBS: phosphate-buffered saline
PPARs: peroxisome proliferator-activated receptors
ROS: reactive oxygen species
STAT3: signal transducer and activator of transcription 3
STE: speckle-tracking echocardiography
S′: peak systolic annular velocity
